# Breeding Behaviors and Kinship in the Endangered Hawaiian Stilt (Ae‘o; *Himantopus mexicanus knudseni*)

**DOI:** 10.1002/ece3.74297

**Published:** 2026-10-13

**Authors:** Melissa R. Price, Kristen C. Harmon, Robert J. Toonen, Evan B. Freel, Madeleine A. Emms, ‘Ale‘alani Dudoit, Coral Loockerman, Arleone Dibben‐Young

**Affiliations:** ^1^ Department of Natural Resources & Environmental Management University of Hawai‘i at Mānoa Honolulu Hawai‘i USA; ^2^ Hawai‘i Institute of Marine Biology, University of Hawai‘i at Mānoa Kāne'ohe Hawai‘i USA; ^3^ Independent Scholar Moloka‘i Hawai‘i USA

**Keywords:** conspecific brood parasitism, cooperative breeding, density‐dependent populations, extra‐pair paternity

## Abstract

Understanding the genetic underpinnings of cooperative and noncooperative breeding behaviors is critical for species of conservation concern. We investigated the genetic relatedness of Hawaiian Stilts (Aeʻo; *Himantopus mexicanus knudseni* ), a threatened subspecies restricted to the Hawaiian Islands, in relation to nesting behavior, given recent behavioral observations of cooperative breeding, potential conspecific brood parasitism, and potential pairing of closely related individuals for nesting. Using RAD sequencing of blood and feather samples, we generated ~6800 SNPs for parentage and relatedness analysis. Observational data documented behaviors including cooperative incubation, helpers‐at‐the‐nest, and potential conspecific brood parasitism, which are rarely reported in Charadriiformes. Parentage, genomic relatedness, and Bayesian phylogenetic analyses revealed instances of conspecific brood parasitism and potential extra‐pair paternity. We also find evidence of inbreeding, with an overall *F*_IS_ of 0.23 and close genetic similarity between some mates. Ecological constraints and limited dispersal opportunities may promote cooperative breeding, conspecific brood parasitism, and inbreeding in Hawaiian Stilts. These results underscore the importance of maintaining and restoring nesting habitat to reduce pressures that may otherwise intensify inbreeding in this recovering population but also highlight the behavioral flexibility and adaptive capacity in this species.

## Graphical abstract

A comparison of behavioral observations with genetic analyses of the Hawaiian Stilt on Molokaʻi revealed some evidence of conspecific parasitism, potential extra‐pair paternity, and inbreeding, although most observed family groups clustered together genetically, suggesting these alternative tactics may be relatively rare. The findings of this study highlight how habitat limitation and constrained dispersal can shape breeding strategies and underscore the conservation value of maintaining and restoring nesting habitat.

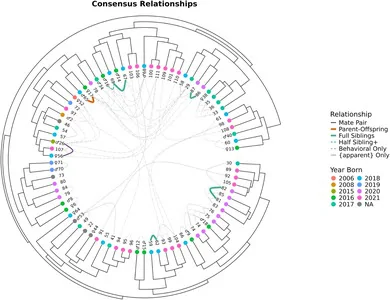

## 1 Introduction

Cooperative breeding, brood parasitism, and other novel breeding behaviors have long been a focus of evolutionary biology, offering a framework to study how relatedness, ecological constraints, and fitness trade‐offs shape animal social systems (Koykka and Wild 2016; Ligon 1983; Lyon and Eadie 2008). Hamilton's rule predicts that cooperative behavior is favored when the inclusive fitness benefits of helping outweigh individual costs (Hamilton 1963), and this theoretical foundation has been widely applied across taxa (Amini et al. 2025; Silva et al. 2025; Turza et al. 2025). However, cooperative behaviors remain relatively undocumented in some avian groups, such as Charadriiformes, where parental care is typically limited to breeding pairs (Ackerman et al. 2014; Adamou et al. 2023), but some instances of cooperative breeding have been observed (Dibben‐Young et al. 2021; Kitagawa 2011).

The Hawaiian Stilt (Aeʻo; *Himantopus mexicanus knudseni* ), a threatened subspecies of the Black‐necked Stilt, presents a unique opportunity to examine the relationship between breeding behaviors and genetic relatedness (Dibben‐Young et al. 2021). This long‐lived, semicolonial waterbird nests in small clusters of nests, with inter‐nest distances as close as 10–20 m (Coleman 1981), and overall population size limited by space and density constraints (Van Rees et al. 2020). Stilts typically nest in sparse, low vegetation or mudflats and are considered primarily monogamous, laying clutches of two to four eggs (Berger 1967), similar to other Charadriiformes (MacLean 1972). Yet recent observations of cooperative incubation by multiple adults, helpers‐at‐the‐nest, and nests containing more than the typical clutch size have been documented on the islands of O‘ahu and Molokaʻi (Dibben‐Young et al. 2021).

Such behaviors in the Hawaiian Stilt could indicate the presence of cooperative breeding, conspecific brood parasitism, or extra‐pair copulations (Dibben‐Young et al. 2021), each of which carries different implications for individual fitness and population dynamics. Cooperative chick defense and helpers‐at‐the‐nest may represent adaptive strategies to cope with limited habitat and high predation pressure (Garcia‐Heras et al. 2024; Harmon, Wehr, and Price 2021; Katayama et al. 2026; Works et al. 2024). Conspecific brood parasitism may increase survival under some circumstances, but potential observations of inbreeding raise concerns for genetic health and long‐term viability (Jackson 1993; McRae 2011). Hawaiian Stilts are currently listed federally as Threatened, but Endangered at the State level, and are considered conservation‐reliant (Van Rees et al. 2022), with invasive mammalian predators (Harmon, Wehr, and Price 2021; Katayama et al. 2026), limited habitat availability due to loss of suitable wetlands (Allen 1998), and sea‐level rise limiting population growth (Harmon, Winter, et al. 2021; Reed et al. 1998). Understanding the relatedness of individuals alongside breeding behavior is therefore essential to guide habitat management and conservation planning.

As these novel breeding behaviors have only recently been documented in Hawaiian Stilts (Dibben‐Young et al. 2021), little is known about the degree to which genetic relatedness is associated with these behaviors. Notably, observations of individually banded birds at a wastewater reclamation facility on Molokaʻi documented a multigenerational family group in which juvenile siblings from previous broods repeatedly assisted their parents in defending nests and chicks against unrelated individuals across multiple breeding seasons (Dibben‐Young et al. 2021). Similarly, a stilt nest containing eight eggs was observed being incubated by one adult male and two different adult females in a wetland on Molokaʻi. These instances raise important questions about whether the cooperative behaviors suggested through observational studies are supported by genetic evidence of parentage and kinship. Here, we paired behavioral observations from Moloka‘i with genetic analyses to explore whether instances of cooperative incubation, conspecific brood parasitism, and other nesting behaviors were supported by parentage and relatedness evidence. Specifically, we asked: (1) Do genetic relationships among breeders and offspring support the behavioral observations of cooperative breeding in Hawaiian Stilts? (2) Is there evidence for conspecific brood parasitism, extra‐pair paternity, or inbreeding? And (3) what are the implications of these behaviors for conservation of this subspecies given current habitat constraints and the potential for restoration and expansion of nesting habitat?

## 2 Methods

### 2.1 Behavioral Observations

The island of Molokaʻi supports populations of Hawaiian Stilts concentrated along the island's south shore. While some marked individuals have been reported moving between the islands of Molokaʻi and Maui, most of the population is resident. However, stilts do move among wetlands on Molokaʻi. Invasive mangrove encroachment has severely reduced available nesting habitat, and remaining wetland habitat is largely unmanaged. As a result, the largest proportion of Hawaiian Stilts on the island consistently nest at the Kaunakakai Wastewater Reclamation Facility (KWRF; 21.0911° N 157.0273° W), as water levels at the facility are maintained at a level preferred by stilts, and potential predators are kept to a minimum due to year‐round trapping and a chain‐linked fence around the facility.

Over the last decade, more than 250 Hawaiian Stilts on Molokaʻi have been marked with unique combinations of colored plastic leg bands by a resident of Molokaʻi (A. Dibben‐Young) with extensive experience and all necessary permits, and behavioral observations of marked individuals have been recorded continuously. In this paper we used data collected from 2012 to 2022 from various wetlands across the south shore of Molokaʻi, including the KWRF. Observational surveys were performed at least once per week year‐round at each site. Band combinations were read using binoculars or spotting scopes from observation points positioned to minimize disturbance. Recorded behaviors included parental care (nest incubation, brooding, escorting chicks), pair interactions (courtship displays, copulations, mate guarding), and social associations (foraging or resting in proximity to specific individuals). For each observed behavior, the identities of all interacting individuals were noted (if known), along with the date, time, location, and context of the interaction. Familial relationships were inferred from repeated observations of marked individuals (Table 1).

**TABLE 1:** Observed behavioral relationships among 91 Hawaiian Stilts (Aeʻo; *Himantopus mexicanus knudsen*) on Molokaʻi.

Sample	USGS band	Sex	Life stage at banding	Hatch year	Behavioral observations
**2**	91438712	M	Adult	2008	Mate pair: sample 52; **Offspring: sample 26**
**6**	91438776	F	Chick	2016	Offspring: sample 77; **Same‐year siblings: sample 8**
**8**	91438778	M	Chick	2016	**Same‐year siblings: sample 6**
**9**	91438779	M	Chick	2016	**Same‐year siblings: sample 18**
10	91438780	—	Chick	2016	None recorded
**12**	91438782	M	Chick	2016	**Same‐year siblings: sample 15**
**13**	91438785	F	Chick	2016	**Offspring:** sample 37, **sample 40**
14	91438786	M	Chick	2016	None recorded
**15**	91438787	M	Chick	2016	**Same‐year siblings: sample 12**
**16**	91438788	M	Chick	2016	Same‐year siblings: sample 17; **Different‐year siblings:** sample 29, sample 32, **sample 67, sample 68, sample 69**
**17**	91438789	F	Chick	2016	**Offspring: sample 65;** Same‐year siblings: sample 16; Different‐year siblings: sample 29, sample 32, sample 67, sample 68, sample 69
**18**	91438790	M	Chick	2016	**Offspring: sample 74, sample 75;** **Same‐year siblings: sample 9**
22	—	—	—	—	None recorded
**26**	91438774	M	Adult	2015	**Father: sample 2; Mother: sample 52;** **Mate pair: sample 56** Offspring: sample 111; Different‐year siblings: sample 72
29	109434718	—	Chick	2017	Same‐year siblings: sample 32; Different‐year siblings: sample 16, sample 17, sample 67, sample 68, sample 69
30	109434719	—	Chick	2017	None recorded
31	109434720	F	Chick	2017	None recorded
32	109434721	F	Chick	2017	Same‐year siblings: sample 29; Different‐year siblings: sample 16, sample 17, sample 67, sample 68, sample 69
**33**	109434722	—	Chick	2017	**Same‐year siblings: sample 35, sample 36, sample 38**
34	109434723	M	Chick	2017	None recorded
**35**	109434724	—	Chick	2017	**Same‐year siblings: sample 33, sample 36, sample 38**
**36**	109434725	—	Chick	2017	**Same‐year siblings: sample 33, sample 35, sample 38**
37	109434726	F	Chick	2017	Mother: sample 13; Same‐year siblings: sample 40
**38**	109434727	F	Chick	2017	**Same‐year siblings: sample 33, sample 35, sample 36**
**40**	109434729	M	Chick	2017	**Mother: sample 13;** Same‐year siblings: sample 37
41	109434730	—	Chick	2017	None recorded
42	109434731	F	Chick	2017	None recorded
43	109434732	M	Chick	2017	None recorded
44	109434733	F	Adult	—	None recorded
46	—	—	—	—	None recorded
47	91438796	M	Chick	2017	None recorded
49	91438798	—	Chick	2017	None recorded
**52**	93492265	F	Adult	2006	Mate pair: sample 2 **Offspring: sample 26, sample 72**
53	109434734	M	Adult	—	None recorded
54	109434735	F	Chick	2017	None recorded
55	109434736	—	Chick	2018	None recorded
**56**	109434737	F	Chick	2018	**Mate pair: sample 26** Offspring: sample 111
57	109434738	F	Chick	2018	None recorded
58	109434739	F	Chick	2018	None recorded
**59**	109434740	F	Chick	2018	**Same‐year siblings: sample 62**
**60**	109434741	M	Chick	2018	**Same‐year siblings: sample 61**
**61**	109434742	M	Chick	2018	**Same‐year siblings: sample 60**
**62**	109434743	M	Chick	2018	**Same‐year siblings: sample 59**
64	109434745	F	Chick	2018	None recorded
**65**	109434746	F	Chick	2018	**Mother: sample 17**
66	109434747	—	Chick	2018	None recorded
**67**	109434748	—	Chick	2018	**Same‐year siblings: sample 68, sample 69;** **Different‐year siblings: sample 16**, sample 17, sample 29, sample 32
**68**	109434749	M	Chick	2018	**Same‐year siblings: sample 67, sample 69;** **Different‐year siblings: sample 16**, sample 17, sample 29, sample 32
**69**	109434750	—	Chick	2018	**Same‐year siblings: sample 67, sample 68;** **Different‐year siblings: sample 16**, sample 17, sample 29, sample 32
70	106406902	F	Chick	2019	None recorded
71	106406903	F	Chick	2019	None recorded
**72**	106406904	—	Chick	2019	**Mother: sample 52;** Different‐year siblings: sample 26
73	—	—	Adult	—	None recorded
**74**	106406905	—	Chick	2020	**Father: sample 18;** **Same‐year siblings: sample 75**
**75**	106406907	M	Chick	2020	**Father: sample 18;** **Same‐year siblings: sample 74**
76	106406908	—	Chick	2020	None recorded
77	106406909	M	Chick	2020	Mother: sample 6
78	106406910	—	Chick	2020	Same‐year siblings: sample 86, sample 87
**79**	106406911	—	Chick	2020	**Same‐year siblings: sample 84**
80	106406912	—	Chick	2020	None recorded
**81**	106406913	—	Chick	2020	**Same‐year siblings: sample 82, sample 83, sample 85**
**82**	106406914	—	Chick	2020	**Same‐year siblings: sample 81, sample 83, sample 85**
**83**	106406915	M	Chick	2020	**Same‐year siblings: sample 81, sample 82, sample 85**
**84**	106406916	F	Chick	2020	**Same‐year siblings: sample 79**
**85**	106406917	—	Chick	2020	**Same‐year siblings: sample 81, sample 82, sample 83**
**86**	106406918	—	Chick	2020	**Same‐year siblings:** sample 78, **sample 87**
**87**	106406919	F	Chick	2020	**Same‐year siblings:** sample 78, **sample 86**
89	106406921	M	Chick	2021	None recorded
90	106406922	M	Chick	2021	Same‐year siblings: sample 91, sample 92
91	106406923	—	Chick	2021	Same‐year siblings: sample 90, sample 92
92	106406924	—	Chick	2021	Same‐year siblings: sample 90, sample 91
**93**	106406925	—	Chick	2021	**Same‐year siblings:** sample 98, **sample 99**
94	106406926	M	Chick	2021	Same‐year siblings: sample 97
**95**	106406927	F	Chick	2021	**Same‐year siblings: sample 96**
**96**	106406928	F	Chick	2021	**Same‐year siblings: sample 95**
97	106406929	—	Chick	2021	Same‐year siblings: sample 94
98	106406930	M	Chick	2021	Same‐year siblings: sample 93, sample 99
**99**	106406931	—	Chick	2021	**Same‐year siblings: sample 93**, sample 98
100	106406932	M	Chick	2021	Same‐year siblings: sample 102
101	—	—	Chick	2021	None recorded
102	106406933	M	Chick	2021	Same‐year siblings: sample 100
103	106406934	—	Chick	2021	Same‐year siblings: sample 104
104	106406935	—	Chick	2021	Same‐year siblings: sample 103
105	—	—	Chick	2021	None recorded
106	106406936	F	Chick	2021	None recorded
107	106406937	—	Chick	2021	Same‐year siblings: sample 110
108	106406938	—	Chick	2021	Same‐year siblings: sample 109
109	106406939	F	Chick	2021	Same‐year siblings: sample 108
110	106406940	—	Chick	2021	Same‐year siblings: sample 107
111	106406941	—	Chick	2021	Father: sample 26; Mother: sample 56
112	106406942	M	Chick	2021	None recorded

*Note:* Relationships are based on long‐term observational surveys of individuals conducted at wetlands across Molokaʻi. Sex and hatch year were determined at time of banding. Sample numbers correspond to genetic sample identifiers used throughout this study. Samples were added to NCBI's Biosample database at BioProject number PRJNA1354480 (BioSamples SAMN52946188 through SAMN52946278). Behavioral relationships in bold are genetically supported—See Table S3 for an expanded version of this table including genomic relatedness and relative positions in a phylogenetic tree. The bold columns are the totals.

### 2.2 Sample Collection

Most individuals were banded as chicks at a minimum of 7 days of age (consistent with permits). Individuals that were banded as adults were captured during the nonbreeding season to avoid nesting disturbance. Blood and/or feather samples were collected from 91 banded individuals, representing ~64% of the most recent population estimate on Molokaʻi (Gorresen et al. 2024). Blood samples were taken by way of venipuncture of the wing, and when possible, a non‐flight feather was collected from adults from the breast of the bird. Whole blood was stored in Longmireʻs solution, and feathers were stored in resealable baggies. All samples were kept frozen at −20°C until processed for DNA extraction and quantification.

Genomic DNA was extracted using the E‐Z 96 Tissue DNA kit (Omega Bio‐Tek Inc.) with two 100 μL elutions in HPLC grade water (concentrated with rotary evaporation if necessary), or the Mag‐Bind Blood and Tissue DNA kit (Omega Bio‐Tek Inc.) with the Kingfisher Flex automated extraction instrument (ThermoFisher Scientific Inc.). Failed extractions were re‐extracted using the column‐based E.Z.N.A. Tissue DNA kit (Omega Bio‐Tek Inc.). Extractions were visualized in a 1.5% agarose gel using 1× TAE buffer, GelRed gel stain (Biotium Inc.), and the Hyperladder 1 kb or 100 bp (Meridian Bioscience Inc.). Most DNA extractions produced a high molecular weight band (> 10 kb), but some samples were partially degraded and were only included in the study if they yielded a smear with at least half the sample above 2.5 kb. Extractions were quantified using either the Qubit dsDNA High Sensitivity kit on the Qubit Flex (ThermoFisher Scientific Inc.) or the AccuBlueTM High Sensitivity dsDNA quantification kit with 8 standards (Biotium Inc), on a SpectraMax M2 microplate reader (Molecular Devices LLC) at λEx/λEm 485/530 nm.

### 2.3 Library Preparation and Sequencing

The ezRAD (Toonen et al. 2013) libraries were prepared for high‐throughput sequencing roughly following the Knapp et al. (2016) protocol. Prior to digestion, degraded samples were treated with AMPure XP purification beads (Beckmann Coulter Inc.) at ~1:0.6 (DNA: beads) ratio to remove smaller DNA fragments. All samples were adjusted to approximately 1 μg of total genomic DNA in 25 μL final volume, where possible. Genomic DNA was then restriction digested using the isoschizomers *Mbo*I and *Sau*3AI (New England BioLabs Inc.), which both cleave at GATC recognition sites. Digests were performed in a 50 μL reaction volume consisting of 25 μL dsDNA (~1 μg), 5 μL NEB CutSmart Buffer (supplied with *Mbo*I), 18 μL HPLC grade or nuclease‐free water, 1 μL *Mbo*I (5 units), and 1 μL *Sau3*AI (5 units) incubated at 37°C for 3 h, before heat deactivation at 65°C for 20 min. Post‐digest, libraries were prepared with variations of the Knapp et al. (2016) protocol (see Table S1 in Supporting Information for details), using either the KAPA Hyper Prep DNA kit (Roche Sequencing and Life Sciences Inc.) or the NEBNext Ultra II DNA Library Prep kit (New England BioLabs Inc.). Briefly, all digests were size‐selected at ~300–700 bp using AMPure XP purification beads (Beckmann Coulter Inc.) manually, or Mag‐Bind TotalPure NGS (Omega Bio‐tek Inc.) purification beads with the Kingfisher Flex automated extraction instrument (ThermoFisher Scientific Inc.), prior to amplification. A total of 47 libraries were sequenced at the Hawaiʻi Institute of Marine Biology (HIMB) Genetic Core Facility on the Illumina MiSeq platform (2 × 150 bp PE). A second pool of 45 libraries was sequenced at University of California Davis' DNA Technologies Core Genome and Biomedical Sciences Facility (GBSF) on the Illumina HiSeq platform (2 × 150 bp PE). One sample (#74) was processed independently through each of the MiSeq and HiSeq library preparation and sequencing runs.

### 2.4 Variant Identification and Filtering

Raw reads were processed using Trim Galore! v0.6.4 (Krueger 2012) for trimming and adapter removal, BWA v0.7.17‐r1188 (bwa‐mem algorithm, Li 2013) for read mapping, and FreeBayes v1.3.8 (Garrison and Marth 2012) for variant calling (see Data Accessibility statement for access to code). Reads were mapped to *de novo* assembled contigs (1.2 Gb, 448,128 contigs, 302 contigs > 50 kb, mean contig length = 2721 bp) generated with SPAdes v3.13.2 (Bankevich et al. 2012) (92% properly paired, 95% mapping quality score “MAPQ” ≥ 30). Mapping statistics were extremely similar when mapping to an alternative congeneric reference genome (also ~1.2 Gb, properly paired reads within ~3% and reads with MAPQ ≥ 30 within 0.35%) so we used the *de novo* assembly which had a slightly higher mapping quality score and therefore a higher chance of identifying specific‐species SNPs. One individual (sample #32) was removed due to excessive missing data (~99%). All variants identified by FreeBayes v1.3.8 (Garrison and Marth 2012) first underwent several basic filters with bcftools v1.20 (Danecek et al. 2021), namely filtering for biallelic SNPs only, with a maximum mean depth of 2000 and at least one non‐reference allele. Remaining SNPs and samples were further filtered for quality down to a final vcf file for further analyses, using VCFtools v0.1.16 (Danecek et al. 2011), vcflib v1.0.10 (Garrison et al. 2022) and bcftools v1.20 (Danecek et al. 2021). Maximum read depth was limited to 500, minimum read depth was 5, maximum per‐site missingness was set at 20%, and maximum per‐individual missingness was set at 30% (see Table S2 in Supporting Information for more details) resulting in a total of 81 samples (80 individuals) and 6837 SNPs. It should be noted that while SNPs were thinned to one per 1000 bp, our longest contigs were over 50 kb, so 295 loci included more than 1 SNP (273 loci had 2 SNPs, 19 loci had 3 SNPs, and 3 loci had 4 SNPs). Inclusion or exclusion of these SNPs had no impact on the final conclusions, so we included all data.

### 2.5 Parentage Analysis and Genomic Relatedness

SNP‐based parentage analysis was performed using the R package {apparent} (Melo and Hale 2019), designed to infer relationships in the absence of prior information such as generation or sex. The vcf file was edited and transposed to the required input file format (genotypes only, missing data as “−/−”). As per the package documentation, a single “key” column was added to provide the limited demographic information we had for the genetic dataset of 81 samples (80 individuals): three observed fathers (“Fa”), five observed mothers (“Mo”), and 27 offspring (“Off”; 28 samples including #74′s duplicate). Offspring were recorded either by observation (7 individuals) or by age (20 individuals hatched in 2021, the last year of sampling, that were not otherwise observed as offspring). It should be noted that sample #26 had both an observed father and observed offspring (Table 1) so was included twice in the input file, once as “Off” and once as “Fa”. All other individuals were tested as a potential parental female or male and offspring (“All”). Though we knew the sex for some of these individuals, this category enabled an individual to be tested as both parent and offspring. This flexibility was necessary given that *H. mexicanus knudseni* individuals typically begin breeding at 2 years of age (Reed et al. 1998), but one individual was observed hatching offspring at 11 months age (pers. obs. ADY). Of all possible parent‐offspring combinations, the {apparent} package (Melo and Hale 2019) aims to identify triads—both parents and their offspring. The minimum number of shared SNPs required for a triad to be considered in the analysis was 500 with alpha of 0.06 (for Dixon outlier test), self‐crossing was not considered, and the optional dyad analysis was also performed (loci = 500, alpha = 0.06, self = FALSE, Dyad = TRUE). Dyad analyses attempted to identify likely single parents for offspring that were not successfully assigned to a pair of parents (e.g., one parent may have been absent from the dataset). All other options used default settings (see Supporting Information for exploration of other settings).

Parentage analysis software perform best for datasets close to Hardy–Weinberg equilibrium with limited inbreeding, clear generational cohorts and allele frequencies that enable clear discrimination between parent‐offspring, sibling, and distant relationships. They tend not to perform as well with inbred study systems such as the one described here, given multiple overlapping generations, blurred genetic distinction among relative groups, and limited priors via observed parent‐offspring relationships. Given the complexity of our study system, we evaluated an alternative R package for parentage inference, {sequoia} (Huisman 2017), but it returned no usable assignments (see Supporting Information) and was not retained for final analyses. Instead, we maximized the value of our combined genetic and behavioral data by taking the output assignments from the {apparent} package, considering these as particularly close relationships, and employing a post‐filtering step—manually inferring the nature of that relationship after considering hatch year. Assignments were reclassified as likely true parent‐offspring relationships, likely full siblings, or half‐siblings+ (which includes all closely related individuals that could not confidently be assigned as parent‐offspring or full siblings). Using VCFtools v0.1.16 (Danecek et al. 2021), we also estimated pairwise genomic relatedness using the A_jk_ estimator (Yang et al. 2010) as an independent metric to evaluate support for behavioral observations and parentage output.

### 2.6 Phylogenetic Analysis

Finally, to complement the close‐kin analyses, we also visualized broader relationships and lineage structure across individuals by generating a Bayesian phylogenetic tree using BEAST v2.7.6 (Bouckaert et al. 2019, 2014). Though we recognize some assumptions are violated when using this method on an inbred population of closely related individuals, it provides an independent comparison as another metric for evaluating the consensus support for (or evidence against) observed relationships. The vcf file was first converted to nexus format using vcf2phylip v2.9 (Ortiz 2019) for importing into BEAUti (within BEAST) to define and generate the required XML file. The GTR substitution model was used based on the lowest AIC (Akaike Information Criterion) score and significant hLRTs (hierarchical likelihood ratio tests) as determined using MrModeltest2 v2.4 (Nylander 2004) and PAUP v4.0a168 (Swofford 2003), which also provided relative substitution rates (AC 0.999, AG 7.644, AT 0.665, CG 0.744, CT 7.913, GT 1.000). Base frequencies were estimated from the data. The analysis was run in BEAST (Bouckaert et al. 2019, 2014) for 10,000,000 MCMC iterations with a sampling frequency of 1000, and the tree prior was set to the default Yule model.

Tracer v1.7.2 (Rambaut et al. 2018) was used to check that the log trace had reached convergence and to determine an appropriate burn‐in period of 2 million. Within BEAST (Bouckaert et al. 2019, 2014), treeannotator was then used to summarize 8001 trees (after designating the 20% burn‐in period) into a single tree file that was visualized as a circular tree with the {ggtree} package (Yu et al. 2017) in R (R Core Team 2021) (see Data Accessibility statement for access to code).

## 3 Results

### 3.1 Genetic Assessment

Sequencing of the original 92 sample libraries (91 individuals) yielded ~913 million raw reads with an average of 10 ± 4.7 million reads per individual. After identifying all called variants, a total of ~11 million SNPs were called, filtered down to 6837 bi‐allelic SNPs and 81 samples (80 individuals) used for analysis. Per‐sample missingness ranged from < 1%–30%, with a mean of 10%. Observed heterozygosity was significantly less than expected, with a mean *F*_IS_ across loci of 0.23 (*H*_*o*_: 0.17, *H*_*e*_: 0.21). Pairwise relatedness ranged from −0.14 to 0.53 with a median of −0.03 (Figure S1 in Supporting Information). The resulting tree from the phylogenetic analysis is shown in Figure S2. The two independent sequencing libraries created from individual #74 (one run on each of the HiSeq and MiSeq platforms) had 5486 of 6837 shared SNP calls and are among the most similar to one another in the tree. Along with consistent alignment of familial relationships across sequencing platforms, this implies minimal batch effects on the data.

This tree was used as a backbone to compare relationships between individuals inferred from behavioral observations (Figure 1) and parentage analysis using {apparent} (Melo and Hale 2019) (Figure 2). A consensus between the two is visualized in Figure 3. Each individual pair that shared a behaviorally‐observed or parentage‐identified relationship was traced back to their most recent common node (MRCN). The number of descendants under these nodes was recorded as a metric of relative position in the tree compared to other sampled individuals. Combining this with pairwise genomic relatedness, a threshold was established to confirm support or rejection of the relationship pairs; comparisons that did not meet thresholds were categorized as indeterminate. Where genomic relatedness was > 0.09, potential pairs always had ≤ 12 descendants under the MRCN, and were considered supported (Tables S3 and S4). This appears to be a suitable threshold when compared to pairwise relatedness across the whole dataset (Figure S1). Where genomic relatedness was ≤ 0.09, counts increased toward the median (Figure S1) and there was an intermediate range for number of descendants under the MRCN. We started to see some higher values (≥ 20 descendants) that were also associated with lower genomic relatedness values, suggesting weak genetic resolution, so potential pairs with genomic relatedness ≤ 0.09 and number of descendants under the MRCN < 80 were considered indeterminate (Tables S3 and S4). Some individuals were filtered out of the dataset, so these relationships were also indeterminate. Considering the threshold for rejection, the highest genomic relatedness value for a pair in the most distant positions in the tree (80 descendants under the MRCN, not counting the repeat library for sample #74) was 0.06. This was a notable point given one sibling pair with this relatedness was relatively close in the tree, sharing 10 descendants under a small node, while another was very distant, sharing 80 descendants under a distant node. We considered these opposed cases to be indeterminate, and pairs with a genomic relatedness value < 0.06 and 80 descendants under the MRCN were considered rejected (Tables S3 and S4). It should be noted from these tables that there was substantial overlap in (supported) relatedness values among relationship categories for both behaviorally observed relationships (parent‐offspring 0.14–0.30; siblings 0.11–0.36) and those identified via parentage analyses (parent‐offspring 0.11–0.33; siblings 0.10–0.23). We note that the use of multiple types of analyses allowed us to infer and reject relationships that any single analysis alone would have missed, and to proceed with more confidence.

**FIGURE 1:**
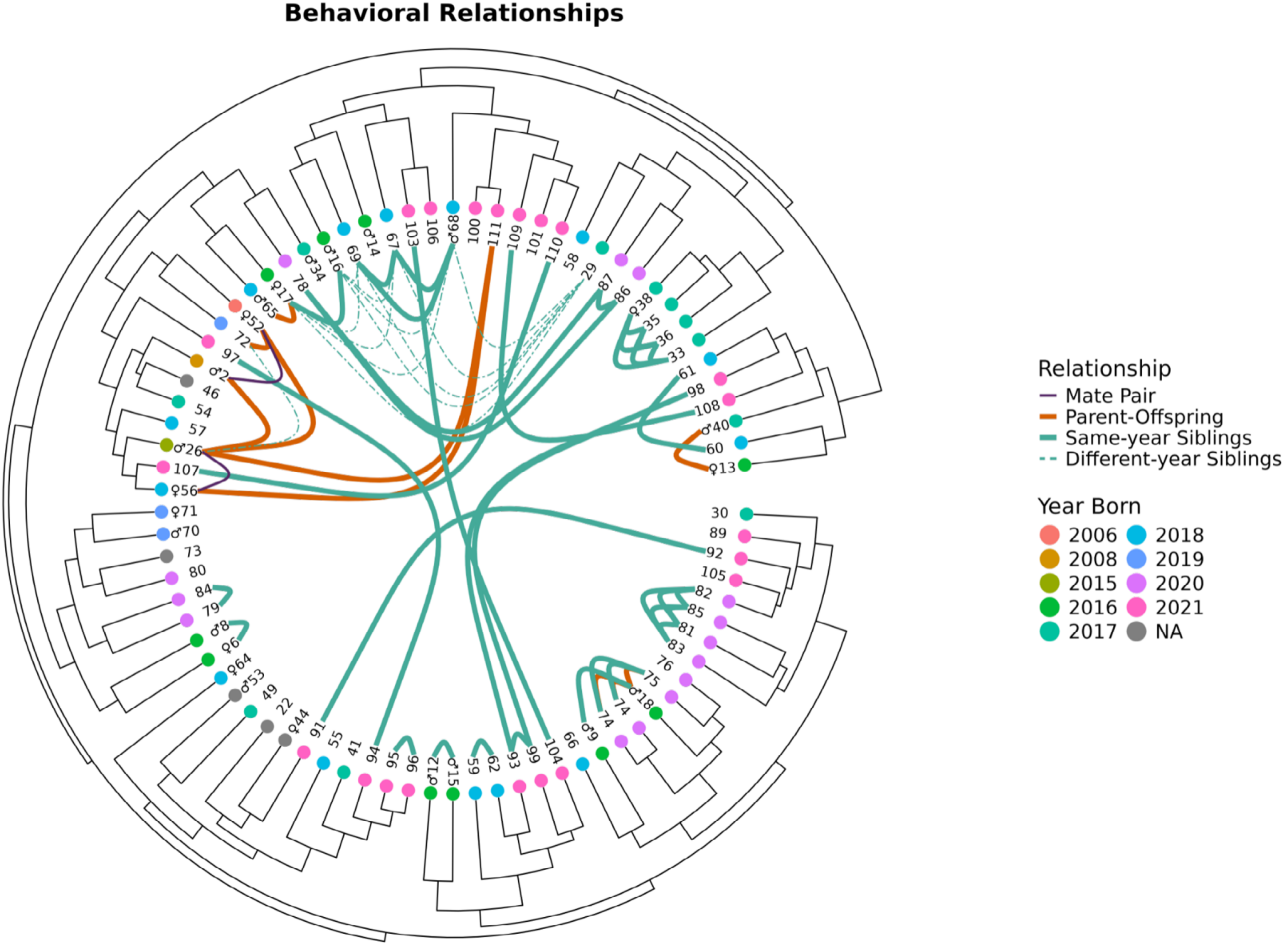
Relationships suggested by visual observations of behavior, mapped onto genetic relatedness. Maximum clade credibility phylogenetic tree from BEAST analysis of Hawaiian Stilt (Ae‘o; *Himantopus mexicanus knudsen*) samples, summarized by treeannotator and visualized with the {ggtree} R package. Tip labels represent individual bird sample IDs. Tip points are colored by hatch year and indicate sex where data are available. Lines connecting tips represent behavioral observations and are colored by relationship (solid purple: Mate pair, solid orange: Parent‐offspring, solid teal: Same‐year siblings, and dashed teal: Different‐year siblings).

**FIGURE 2:**
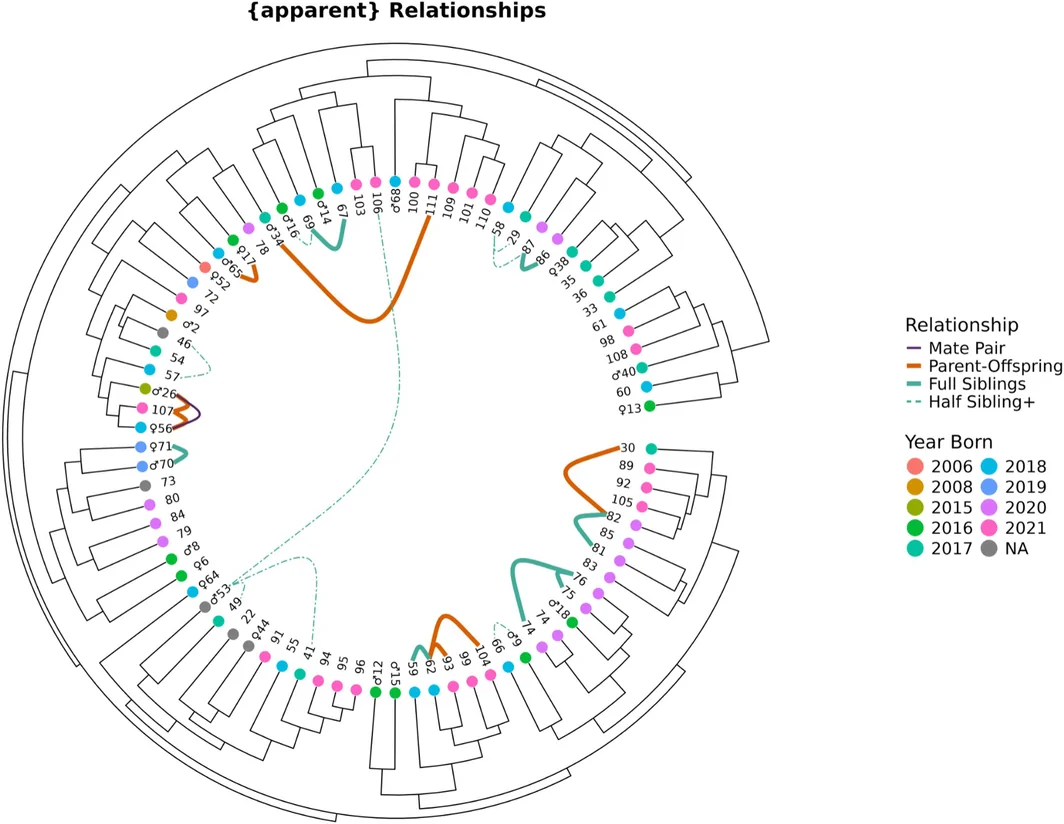
Relationships supported by parentage analyses, mapped onto genetic relatedness. Maximum clade credibility phylogenetic tree from BEAST analysis of Hawaiian Stilt (Ae‘o; *Himantopus mexicanus knudsen*) samples, summarized by treeannotator and visualized with the {ggtree} R package. Tip labels represent individual bird sample IDs. Tip points are colored by hatch year and indicate sex where data are available. Lines connecting tips represent relationships as assigned by the {apparent} R package but are colored by the reclassified relationship, depending on hatch year, corrected from the default parent‐offspring relationship (solid purple: Mate pair, solid orange: Parent‐offspring, solid teal: Full siblings, and dashed teal: Half‐siblings+ i.e., unable to be confirmed as likely true parent‐offspring or siblings).

**FIGURE 3:**
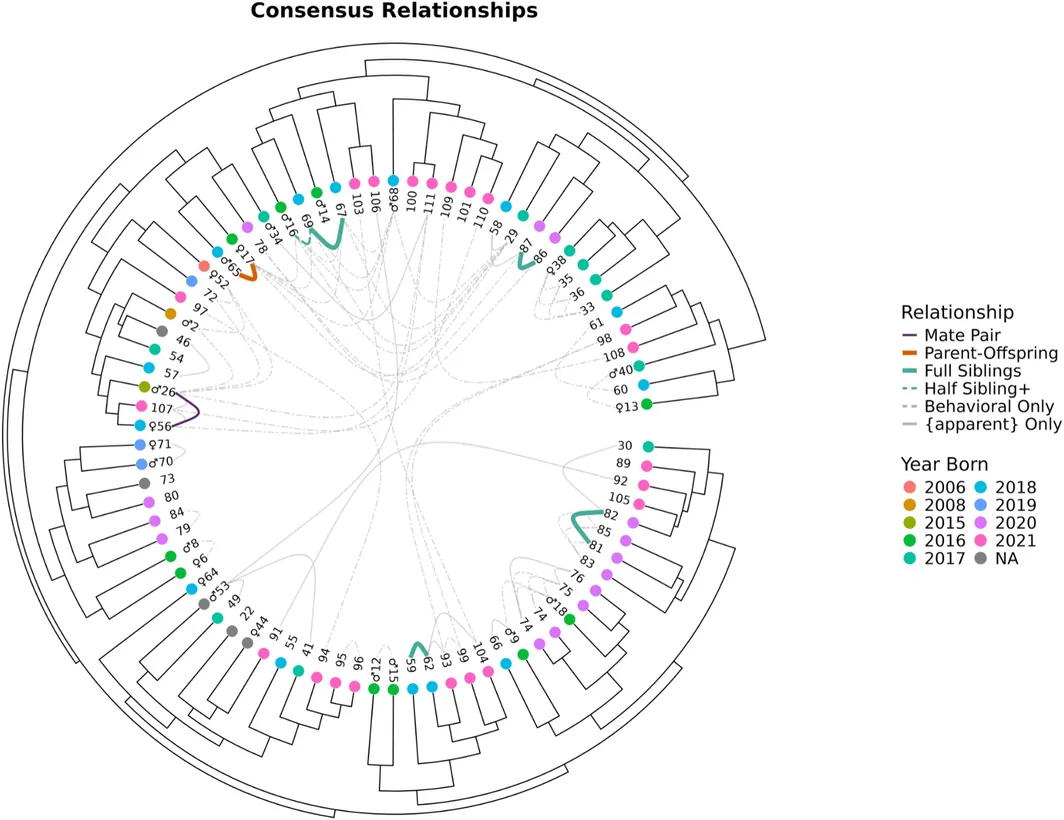
Relationships supported by both parentage analyses and behavioral data. Maximum clade credibility phylogenetic tree from BEAST analysis of Hawaiian Stilt (Ae‘o; *Himantopus mexicanus knudsen*) samples, summarized by treeannotator and visualized with the {ggtree} R package. Tip labels represent individual bird sample IDs. Tip points are colored by hatch year and indicate sex where data are available. Lines connecting tips represent relationships as assigned by both behavioral observations and parentage analysis via the {apparent} R package. Lines are colored by relationship when supported by both behavioral observations and parentage analysis (solid purple: Mate pair, solid orange: Parent‐offspring, solid teal: Full siblings, and dashed teal: Half‐siblings+ i.e., unable to be confirmed as likely true parent‐offspring or siblings). Faint gray lines represent relationships only supported by one of either behavioral or parentage methodology (dashed gray lines only supported by behavioral observations, and solid gray lines only supported by parentage analysis).

### 3.2 Behavioral Observations

Behaviorally‐observed relationships included 21 sibling groups; 15 groups being sibling pairs (14 hatched in the same year and one hatched across different years) and six groups consisting of more than two siblings (five groups of three to four siblings hatched in the same year and one group of six siblings hatched across different years). Eight parents and seven parent‐offspring groups involving up to three individuals were observed: two involved two parents with one offspring, three were one parent with one offspring, and the final two were one parent with two offspring each (Tables 1 and 2).

**TABLE 2:** A summary of the number of individual pairwise relationships in groups of Hawaiian Stilts (Ae‘o; *Himantopus mexicanus knudsen*), either behaviorally observed or identified by parentage analysis, that were supported, rejected, or indeterminate following genetic analyses of a SNP dataset.

	Behavioral data				Parentage data					Consensus
	# Observed	Supported	Rejected	Indeterminate	# Significant	Reclassification based on hatch year	Supported	Rejected	Indeterminate	
	**21 siblings groups**					**15 sibling dyads**				
Sibling pairs hatched in same year	**14**	8	2^[CBP/EPP]^	4		**4**	4	0	0	1
Sibling pairs part of 5 groups (3–4 ind) hatched in same year	**21**	14	3^[CBP/EPP]^	4		**2**	2	0	0	2
Sibling pairs hatched across different years	**1**	0	0	1		**3**	3	0	0	0
Sibling pairs part of 1 group (6 ind) hatched across different years	**16** (diff years) **+ 5** (same year)	3 (diff years) + 3 (same year)	4 (diff years) + 1 (same year^[CBP/EPP]^)	9 (diff years) + 1 (same year)		**1** (diff years) **+ 1** (same year)	1 (diff years) + 1 (same year)	0	0	1 (diff years) + 1 (same year)
Pairs with a missing hatch year						**4**	1	1	2	0
	**7 parent‐offspring groups**					**6 parent‐offspring triads/dyads**				
Pairs in 2 parents—1 offspring groups	**4**	2	1^[CBP]^ + 1^[CBP/EPP]^	0	**2**	**2**	2	0	0	0
Pairs in 1 parent—1 offspring groups	**3**	2	0	1	**20**	**5**	4	1^[CBP/EPP]^	0	1
Pairs in 1 parent—2 offspring groups	**4**	3	0	1						0
**Mate pair relations**										
Mate pairs	**2**	1^[IB]^	0	1	**1**	**1**	1^[IB]^	0	0	1^[IB]^

*Note:* The threshold for support was based on a combination of pairwise genomic relatedness and position in a phylogenetic tree. The 23 dyads (one parent—one offspring pair; includes 3 repeats; alpha = 0.06, Dixon outlier test) identified in the parentage analysis were reclassified based on hatch year. The consensus column includes genetically supported, behaviorally observed pairs of individuals also identified (and supported) in the parentage analysis. Values denoted with superscript abbreviations summarize relationships used as evidence for conspecific brood parasitism [CBP], conspecific brood parasitism or extra‐pair paternity [CBP/EPP], and inbreeding [IB]. The bold columns are the totals.

Just over half of all relationships between individuals inferred from behavioral observations (Figure 1) were supported by the genetic data (51%), with only 17% rejected and the rest indeterminate (Tables 1 and 2, Table S3). Of 40 sibling‐pair relationships involving observed siblings hatched in the same year, only six cases were rejected and nine were indeterminate. However, there were examples where individuals behaviorally observed as siblings or parent‐offspring were more genetically distant than expected. For example, fewer observed relationships were supported among the sibling group that hatched across different years (Table 2). One of the eight behaviorally observed parents (#6) could not be confirmed genetically due to the observed offspring being filtered from the dataset during data quality checks, and two were rejected as likely parents given low genomic relatedness and their divergent positions in the tree (a behavioral father (#26) and a behavioral mother (#56) to an observed offspring (#111); Table S3). Interestingly, this case also involved one of only two observed mate pairs (#26, #56) which were also found to be very closely related to each other (Tables S3 and S4, Table 2), providing evidence of inbreeding among related individuals in this population.

### 3.3 {Apparent} Parentage Analysis

Parentage analysis identified one significant triad using 4415 shared SNPs. Shared SNPs are not reported for the dyad analyses, but 5121 SNPs (of 6837 in the filtered dataset) had a minor allele frequency > 0.05 and likely contributed to this inference, which found 23 significant dyads (reduced to 20, with three repeated dyads). This parentage approach detects close relationships, but prior to reclassification, 15 of the 20 putative parent‐offspring dyad assignments were very unlikely when considering the observed/recorded hatch years of the individuals. For example, three dyads were reported twice showing each individual alternatively as the offspring and the parent, two dyads identified a younger individual as the parent and an older individual as the offspring, and seven dyads were made up of individuals that hatched in the same year. The software struggled to determine siblings from parent‐offspring pairs in this population, but manual reclassification based on observed hatch year likely recovers true parent‐offspring relationships, full siblings, or half‐siblings+. Parentage approaches are likely to struggle for any small, isolated and inbred population, highlighting the value of including multiple genetic analyses and other demographic and behavioral data as independent lines of evidence.

Of the 20 significant dyads (see Table S4 in Supporting Information), five were recorded as likely true parent/offspring pairs as confirmed by their observed hatch years. Seven dyads comprising individuals hatched in the same year were recorded as full siblings. Two dyads with parent/offspring roles reversed relative to hatch years, three repeated dyads assigning individuals to both parent and offspring roles, and three where missing hatch year information precluded manual assignment were all recorded as half‐siblings+.

When aligning relationships between individuals inferred from our {apparent} (Melo and Hale 2019) output with the phylogenetic tree and genomic relatedness (Figure 2, Table S4), the significant triad and most of the dyads were supported. This included the relationship between the mate pair in the triad, a consensus relationship (also behaviorally observed) supported by the genetic data. Two dyads were deemed indeterminate due to a relatively low genomic relatedness value (both half‐siblings+). Two were rejected as genetically distant individuals (one parent‐offspring dyad representing a paternal relationship and one half‐siblings+ dyad). Thus we identified six genetically supported parents via parentage analysis (of seven after reclassification).

### 3.4 Consensus Tree

Only seven relationships between 80 individuals were identified with both behavioral observations and genetic parentage analyses and supported by pairwise genomic relatedness and position in the phylogenetic tree. These are noted in Table S4 and highlighted in color in the consensus tree (Figure 3). Five were likely full sibling pairs, one was likely a true parent‐offspring relationship, and one appears to be a very closely related mate pair (of the same relative magnitude as these sibling and parent‐offspring relationships).

## 4 Discussion

This study was the first to evaluate whether genetic evidence was consistent with behavioral observations of cooperative and parasitic breeding behaviors in the Hawaiian Stilt. Our study found genetic evidence consistent with conspecific brood parasitism, potential extra‐pair paternity, and inbreeding. Thus, Hawaiian Stilts may employ multiple reproductive strategies, depending on ecological and social contexts, similar to rare observations of cooperative breeding in the Black‐winged Stilt (Kitagawa 2011).

In cases where the observed behavioral mother was not the genetic parent of an offspring, the genetic mother may have her own nest and be laying her eggs in the nest of another nesting pair. Alternatively, she may be a cooperative breeder in the nest in which she laid the eggs, participating in breeding but not laying the majority of the eggs (Eadie and Lyon 2011). Our evidence suggests conspecific brood parasitism, because the observed maternal relationship between #56 and #111 is rejected based on the genetic data, and cooperative breeding usually involves individuals more closely related to one another than the rest of the population. To our knowledge, this is the first genetic confirmation of conspecific brood parasitism in a stilt species. The rejection of the observed paternal relationship between #26 and #111 is further evidence in this mate pair case, though extra‐pair paternity (occurring concurrently) cannot be ruled out. Another paternal relationship (identified via parentage analysis; #34 and #111) and the five cases of observed nestmates (i.e., potential siblings) hatched in the same year, that were all rejected based on genetic data, provide evidence for either conspecific brood parasitism or extra‐pair paternity. However, more data would be required to discriminate between these two explanations.

With large populations concentrated at only a few sites on the island of Molokaʻi, opportunities for dispersal are limited, and density‐dependent factors likely exacerbate competition for nesting space (Van Rees et al. 2020). Environmental constraints that result in localized dispersal may result in noncooperative breeding behaviors that harm the fitness of conspecific or heterospecific nesting individuals (Flatrès and Wild 2025). Egg destruction by Hawaiian Stilt conspecifics and other waterbird species has been observed in wetlands with a high density of nesting birds (Works et al. 2024, pers. obs. A. Dibben‐Young, pers. obs. K. Harmon), and conspecific brood parasitism may also result in lower survival of the host parent chicks, for example if competition for food resources is high after hatching, or perhaps due to physical limitations in stilts that constrain successful brooding to four eggs (Kitagawa 2011).

In contrast, cooperative behaviors have also been previously observed, such as nests with three individuals participating in brooding duties, mobbing behavior in which 10 adult individuals joined in defensive nest behaviors when one nest was approached by a potential predator (Botet‐Rodriguez et al., under review), and at least one case of cooperative chick care in which five adults surrounded large groups of chicks (Dibben‐Young et al. 2021). While we were not able to explore the genetic relatedness of individuals participating in these activities, it is clear that both cooperative and noncooperative breeding behaviors are occurring in the same populations of Hawaiian Stilts. Further studies are needed to explore the role of environmental factors such as predation rates (McRae 2024), as well as the influence of saturation of breeding territories or nest sites, evolutionary trade‐offs for each strategy, and species responses to expanding habitat as restoration activities occur. Creating additional nesting areas for this recovering species would reduce ecological constraints on recovering populations (Reed et al. 2011; Underwood et al. 2013).

Genetic evidence of inbreeding was also detected in this study, with a moderate *F*_IS_ (0.23) indicating a heterozygote deficit, and one of the only two observed mate pairs (#26, #56) being closely related, potentially even siblings. This relationship was strongly supported as one of only seven consensus relationships in the dataset (genetically supported, behaviorally observed, and identified by the parentage analysis). Evidence of inbreeding is concerning given the restricted range of the subspecies and relatively small population size of less than 1800 individuals across the Hawaiian Islands, and less than 200 individuals on the island of Moloka‘i where this study occurred (Paxton et al. 2021). If inbreeding increases, it could reduce fitness (Hagen et al. 2011), exacerbate susceptibility to disease, and lead to the loss of alleles, which are issues of particular importance for threatened taxa facing rapid environmental change associated with habitat loss due to sea level rise (Harmon, Winter, et al. 2021). However, we note that other endangered stilt species have maintained genetic diversity despite dramatic bottlenecks (Forsdick et al. 2025), offering hope for the Hawaiian Stilt as it recovers.

We note that relatively few parents were identified in our study, limiting our inferences. While recent reviews have found parentage analyses to be reliable, particularly in assigning maternal parents (Thow et al. 2022), accurate assignment is particularly challenging in populations with very low population genetic structure or a high degree of relatedness due to inbreeding (Christie et al. 2017). Given fewer than 200 individuals on the island of Molokaʻi where this study occurred (Paxton et al. 2021), and significant reduction in heterozygosity and inbreeding coefficient (*F*_IS_), we suspect that a relatively high degree of relatedness within populations may be the norm. However, Hawaiian Stilts do move among islands (Engilis Jr. and Pratt 1993; Reed et al. 1998), despite relatively high fidelity to individual wetlands (Paxton et al. 2023), and this movement may contribute to maintaining genetic diversity in small island populations. More genetic analyses across the Hawaiian Islands are needed to determine the magnitude of exchange among islands, and whether inbreeding is a cause for concern. We were also unable to test for helpers‐at‐nest. Seven observed parent‐offspring relationships and six parent‐offspring pairs from the parentage analysis were genetically supported, but we were unable to discern whether they were biological parents or closely‐related helpers. Thus, a further avenue for inquiry would be to test a larger dataset of observed parents to unambiguously determine relatedness to offspring.

Our study demonstrates the value of combining behavioral observations with genomic approaches to resolve questions of breeding system complexity in threatened taxa, particularly those with limited genetic diversity and potential inbreeding. For Hawaiian Stilts, such integrated methods allow us to distinguish between cooperative behaviors that enhance population resilience and noncooperative strategies that may increase vulnerability by decreasing overall nesting success. As conservation efforts continue, ongoing monitoring of genetic relatedness and reproductive strategies will be essential to ensure long‐term recovery of the Hawaiian Stilt.

## Author Contributions

**Melissa R. Price:** conceptualization (equal), methodology (equal), project administration (equal), resources (equal), visualization (equal), writing – original draft (equal), writing – review and editing (equal). **Kristen C. Harmon:** conceptualization (equal), formal analysis (equal), project administration (equal), visualization (equal), writing – original draft (equal), writing – review and editing (equal). **Robert J. Toonen:** conceptualization (equal), formal analysis (equal), funding acquisition (equal), methodology (equal), project administration (equal), visualization (equal), writing – review and editing (equal). **Evan B. Freel:** formal analysis (equal), methodology (equal), visualization (equal), writing – review and editing (equal). **Madeleine A. Emms:** formal analysis (equal), methodology (equal), visualization (equal), writing – review and editing (equal). **‘Ale‘alani Dudoit:** formal analysis (equal), methodology (equal), visualization (equal), writing – review and editing (equal). **Coral Loockerman:** formal analysis (equal), methodology (equal), writing – review and editing (equal). **Arleone Dibben‐Young:** conceptualization (equal), investigation (equal), methodology (equal), resources (equal), writing – review and editing (equal).

## Funding

This work was supported by Nēnē O Molokaʻi. NOAA Office of Sea Grant, Department of Commerce, NA18OAR4170108.

## Conflicts of Interest

The authors declare no conflicts of interest.

## Supporting information


**Table S1:** ez‐RAD library preparation steps for Hawaiian Stilt (Ae‘o; *Himantopus mexicanus knudsen*) samples from extracted DNA to final pools for sequencing. The two runs were variations of the Knapp et al. (2016) protocol, using either the KAPA Hyper Prep DNA kit and AMPure XP beads (run 1) or the NEBNext Ultra II DNA Library Prep kit and Mag‐Bind TotalPure NGS (Omega Bio‐tek Inc.) beads (run 2).
**Table S2:** Relevant filters applied to ~11 million variants (identified by FreeBayes), using bcftools, VCFtools, and vcflib. See relevant package documentation for filter descriptions. The original Hawaiian Stilt (Ae‘o; *Himantopus mexicanus knudsen*) reads were mapped to *de novo* assembled contigs.
**Table S3:** Observed behavioral relationships among 91 Hawaiian Stilts (Ae‘o; *Himantopus mexicanus knudsen*) on Molokaʻi. Relationships are based on long‐term observational surveys of individuals conducted at wetlands across Molokaʻi. Sex and hatch year were determined at time of banding. Sample numbers correspond to genetic sample identifiers used throughout this study. Samples were added to NCBI's Biosample database at BioProject number PRJNA1354480 (BioSamples SAMN52946188 through SAMN52946278). The right side of the table provides genetic information—pairwise genomic relatedness (GR) and relative position in a phylogenetic tree—which together provided a threshold for whether a pairwise relationship was supported (S, also in bold), rejected (R), or indeterminate (I). Superscript abbreviations highlight relationships used as evidence for conspecific brood parasitism [CBP], conspecific brood parasitism or extra‐pair paternity [CBP/EPP], and inbreeding [IB]. NA values occur where an individual was filtered from the genetic dataset.
**Table S4:** Significant triad (two parents and one offspring) and dyad (one parent and one offspring) outputs following parentage analysis with {apparent} on a breeding population of Hawaiian Stilt (Ae‘o; *Himantopus mexicanus knudsen*) individuals (GD: Gower Dissimilarity metric, GDM: mean GD, GDCV: coefficient of variation among the pairwise GD's). The central section of the table provides extra information about the individuals based on observed behavioral data. Note that ‘code’ represents the reclassified relationship, depending on hatch year, corrected from the default parent‐offspring relationship output by {apparent} (P/O: likely true parent‐offspring, FS: likely full siblings, and HS+: half‐siblings+ i.e., unable to be confirmed as likely true parent‐offspring or siblings). The behavioral info column highlights consensus relationships. The right side of the table provides additional genetic information—pairwise genomic relatedness (GR) and relative position in a phylogenetic tree—which together provided a threshold for whether a relationship was supported, rejected, or indeterminate. Values denoted with superscript abbreviations summarize relationships used as evidence for conspecific brood parasitism or extra‐pair paternity [CBP/EPP], and inbreeding [IB]. Italicized rows highlight repeat dyads with both parent and offspring roles suggested for each individual.
**Figure S1:** Distribution of pairwise genomic relatedness values for a filtered dataset of 81 Hawaiian Stilt (Ae‘o; *Himantopus mexicanus knudsen*) samples, calculated using Yang's A_jk_ estimator in VCFtools.
**Figure S2:** Maximum clade credibility phylogenetic tree from BEAST analysis of Hawaiian Stilt (Ae‘o; *Himantopus mexicanus knudsen*) samples, summarized by treeannotator and visualized with the {ggtree} R package. Tip labels represent individual bird sample IDs and indicate sex where data are available.

## Data Availability

Sequence data are openly accessible via NCBI's Sequence Read Archive (SRA) at BioProject number PRJNA1354480 (SRA Accession numbers SRR36109226 through SRR36109136; BioSamples SAMN52946188 through SAMN52946278). The bash and R code used for raw read processing, mapping, variant calling, and data visualization is openly available on Zenodo (10.5281/zenodo.20337032).
