# Supplementary material for: Breeding Behaviors and Kinship in the Endangered Hawaiian Stilt (Ae‘o; Himantopus mexicanus knudseni)
Source: Ecol Evol. 2026 Oct 13;16(10):e74297. doi: 10.1002/ece3.74297 (PMC13659274; doi:10.1002/ece3.74297)
Supplement: Supplementary file 1 [file ECE3-16-e74297-s001.docx]

## Supplementary Information

{apparent} package settings

Increasing MaxIdent to encompass all possible triads did not alter the results, so we proceeded without specifying a value (maintaining the default at 0.1) to reduce computation time. We also experimented with other alpha values and while most dyads were maintained, a larger number of dyads were identified that were rejected based on other genetic data. Thus, we decided to proceed with further analyses using the alpha value of 0.06.

{sequoia} package settings

The filtered vcf was converted to .ped file using plink2 and was used as input alongside available life history data for the samples. Parameter settings used beyond defaults included CalcLLR = T, Err = 0.005, Module = 'ped', StrictGenoCheck = F and UseAge = "extra”. No parents or grandparents were identified and nine sibling relationships were marked as likely related assuming only dummy parent assignment.

Supplementary Tables

**Table S1.** ez-RAD library preparation steps for Hawaiian Stilt (Ae‘o; *Himantopus mexicanus knudsen*) samples from extracted DNA to final pools for sequencing. The two runs were variations of the Knapp et al. (2016) protocol, using either the KAPA Hyper Prep DNA kit and AMPure XP beads (run 1) or the NEBNext Ultra II DNA Library Prep kit and Mag-Bind TotalPure NGS (Omega Bio-tek, Inc.) beads (run 2).

| **RUN 1** | | **RUN 2** | |
| --- | --- | --- | --- |
| **All cleanups used AMPure XP beads** | | **All cleanups used Mag-Bind TotalPure NGS beads** | |
| Extraction cleanup: 0.6X beads | Knapp et al 2016 | NA | |
| Concentration adjustment and digest |  | Concentration adjustment and digest | Knapp et al 2016 |
| Post-digest cleanup; 1.8X beads |  | NA | |
| NA | | Size selection | ‘Illumina library prep’ section of Knapp et al 2016 |
| End-repair and A-tailing | ‘Kapa library prep’ section of Knapp et al 2016, using KAPA Hyper Prep DNA kit | NEBNext End Prep | NEBNext Ultra II DNA Library Prep kit protocol |
| Adapter ligation |  | Adapter ligation |  |
| Post-ligation cleanup: 0.8X beads |  | Post-ligation cleanup: 1.8X beads | |
| Size selection |  | NA | |
| Library Amplification |  | PCR Enrichment of Adaptor-ligated DNA | NEBNext Ultra II DNA Library Prep kit protocol |
| Post-amplification cleanup: 1X beads |  | Post-amplification cleanup: 1.8X beads | |
| Validate libraries and pool | | Validate libraries and pool | |
| NA | | Sequencing facility cleaned pool with 0.8X (KapaPure) beads | |

**Table S2.** Relevant filters applied to ~11 million variants (identified by FreeBayes), using bcftools, VCFtools, and vcflib. See relevant package documentation for filter descriptions. The original Hawaiian Stilt (Ae‘o; *Himantopus mexicanus knudsen*) reads were mapped to *de novo* assembled contigs.

| **bcftools** | **VCFtools** | **vcflib (VCFfilter)** |
| --- | --- | --- |
| view --max-alleles 2 | (--missing-site)  --max-missing 0.8 | MQM & MQMR > 30 |
| view -i ‘MEAN(FORMAT/DP)<=2000’ | --mac 2 | MQM / MQMR > 0.75  & MQM / MQMR < 1.25 |
| view -v snps | --min-meanDP 5 | QUAL / DP > 0.25 |
| view -c 1 | --max-meanDP 500 | PAIRED > 0.05 & PAIREDR > 0.05  & PAIREDR / PAIRED < 1.75  & PAIREDR / PAIRED > 0.25 |
| view -g hom | --thin 1000 | NS > 44.5 |
| filter -e ‘strlen(REF)!=1 \|\| strlen(ALT)!=1’ | (--hardy for H_o_ and --freq for MAF, per locus)  --positions list_SNPs_het0.4.txt | DP > 30 |
|  | (--missing-indv) --remove list_samples_fmiss0.3.txt | QUAL > 20 |
|  |  | TYPE = snp |
|  |  | AO > 2 |

**Table S3.** Observed behavioral relationships among 91 Hawaiian Stilts (Ae‘o; *Himantopus mexicanus knudsen*) on Molokaʻi. Relationships are based on long-term observational surveys of individuals conducted at wetlands across Molokaʻi. Sex and hatch year were determined at time of banding. Sample numbers correspond to genetic sample identifiers used throughout this study. Samples were added to NCBI’s Biosample database at BioProject number PRJNA1354480 (BioSamples SAMN52946188 through SAMN52946278). The right side of the table provides genetic information - pairwise genomic relatedness (GR) and relative position in a phylogenetic tree - which together provided a threshold for whether a pairwise relationship was supported (S, also in bold), rejected (R), or indeterminate (I). Superscript abbreviations highlight relationships used as evidence for conspecific brood parasitism [CBP], conspecific brood parasitism or extra-pair paternity [CBP/EPP], and inbreeding [IB]. NA values occur where an individual was filtered from the genetic dataset.

| **Sample** | **USGS Band** | **Sex** | **Life Stage**  **at Banding** | **Hatch Year** | **Behavioral Observations** | **GR** | **# descendents under most recent common node** | **Result (Supported, Rejected or Indeterminate)** |
| --- | --- | --- | --- | --- | --- | --- | --- | --- |
| **2** | 91438712 | M | Adult | 2008 | Mate pair: sample 52;  **Offspring: sample 26** | -0.1;  **0.17** | 10;  **7** | I;  **S** |
| **6** | 91438776 | F | Chick | 2016 | Offspring: sample 77;  **Same-year siblings: sample 8** | NA;  **0.13** | NA;  **2** | I;  **S** |
| **8** | 91438778 | M | Chick | 2016 | **Same-year siblings: sample 6** | **0.13** | **2** | **S** |
| **9** | 91438779 | M | Chick | 2016 | **Same-year siblings: sample 18** | **0.14** | **6** | **S** |
| 10 | 91438780 | — | Chick | 2016 | None recorded | - | - | - |
| **12** | 91438782 | M | Chick | 2016 | **Same-year siblings: sample 15** | **0.15** | **2** | **S** |
| **13** | 91438785 | F | Chick | 2016 | **Offspring:** sample 37, **sample 40** | NA, **0.20** | NA, **6** | I, **S** |
| 14 | 91438786 | M | Chick | 2016 | None recorded | - | - | - |
| **15** | 91438787 | M | Chick | 2016 | **Same-year siblings: sample 12** | **0.15** | **2** | **S** |
| **16** | 91438788 | M | Chick | 2016 | Same-year siblings: sample 17;  **Different-year siblings:** sample 29, sample 32,  **sample 67, sample 68, sample 69** | 0.01;  0.05, NA,  **0.13, 0.16, 0.15** | 80;  20, NA,  **6, 12, 2** | R^[CBP/EPP]^;  I, I,  **S, S, S** |
| **17** | 91438789 | F | Chick | 2016 | **Offspring: sample 65;**  Same-year siblings: sample 16;  Different-year siblings: sample 29, sample 32,  sample 67, sample 68, sample 69 | **0.30;**  0.01;  -0.08, NA,  -0.01, -0.00, 0.03 | **2;**  80;  80, NA,  80, 80, 80 | **S;**  R^[CBP/EPP]^;  R, I,  R, R, R |
| **18** | 91438790 | M | Chick | 2016 | **Offspring: sample 74, sample 75;**  **Same-year siblings: sample 9** | **0.14/0.17, 0.19;**  **0.14** | **4, 5;**  **6** | **S, S;**  **S** |
| 22 | — | — | — | — | None recorded | - | - | - |
| **26** | 91438774 | M | Adult | 2015 | **Father: sample 2; Mother: sample 52;**  **Mate pair: sample 56**  Offspring: sample 111;  Different-year siblings: sample 72 | **0.17; 0.14;**  **0.20;**  -0.06;  0.06 | **7; 10;**  **3;**  80;  10 | **S; S;**  **S^[IB]^;**  R^[CBP/EPP]^;  I |
| 29 | 109434718 | — | Chick | 2017 | Same-year siblings: sample 32;  Different-year siblings: sample 16, sample 17,  sample 67, sample 68, sample 69 | NA;  0.05, -0.08,  0.08, 0.02, 0.09 | NA;  20, 80,  20, 20, 20 | I;  I, R,  I, I, I |
| 30 | 109434719 | — | Chick | 2017 | None recorded | - | - | - |
| 31 | 109434720 | F | Chick | 2017 | None recorded | - | - | - |
| 32 | 109434721 | F | Chick | 2017 | Same-year siblings: sample 29;  Different-year siblings: sample 16, sample 17,  sample 67, sample 68, sample 69 | NA | NA | I |
| **33** | 109434722 | — | Chick | 2017 | **Same-year siblings: sample 35, sample 36, sample 38** | **0.23, 0.28, 0.24** | **4, 4, 4** | **S, S, S** |
| 34 | 109434723 | M | Chick | 2017 | None recorded | - | - | - |
| **35** | 109434724 | — | Chick | 2017 | **Same-year siblings: sample 33, sample 36, sample 38** | **0.23, 0.31, 0.28** | **4, 3, 2** | **S, S, S** |
| **36** | 109434725 | — | Chick | 2017 | **Same-year siblings: sample 33, sample 35, sample 38** | **0.28, 0.31, 0.31** | **4, 3, 3** | **S, S, S** |
| 37 | 109434726 | F | Chick | 2017 | Mother: sample 13;  Same-year siblings: sample 40 | NA | NA | I |
| **38** | 109434727 | F | Chick | 2017 | **Same-year siblings: sample 33, sample 35, sample 36** | **0.24, 0.28, 0.31** | **4, 2, 3** | **S, S, S** |
| **40** | 109434729 | M | Chick | 2017 | **Mother: sample 13;**  Same-year siblings: sample 37 | **0.2;**  NA | **6;**  NA | **S;**  I |
| 41 | 109434730 | — | Chick | 2017 | None recorded | - | - | - |
| 42 | 109434731 | F | Chick | 2017 | None recorded | - | - | - |
| 43 | 109434732 | M | Chick | 2017 | None recorded | - | - | - |
| 44 | 109434733 | F | Adult | — | None recorded | - | - | - |
| 46 | — | — | — | — | None recorded | - | - | - |
| 47 | 91438796 | M | Chick | 2017 | None recorded | - | - | - |
| 49 | 91438798 | — | Chick | 2017 | None recorded | - | - | - |
| **52** | 93492265 | F | Adult | 2006 | Mate pair: sample 2  **Offspring: sample 26, sample 72** | -0.1;  **0.14, 0.10** | 10;  **10, 2** | I;  **S, S** |
| 53 | 109434734 | M | Adult | — | None recorded | - | - | - |
| 54 | 109434735 | F | Chick | 2017 | None recorded | - | - | - |
| 55 | 109434736 | — | Chick | 2018 | None recorded | - |  |  |
| **56** | 109434737 | F | Chick | 2018 | **Mate pair: sample 26**  Offspring: sample 111 | **0.20;**  -0.05 | **3;**  80 | **S^[IB]^;**  R^[CBP]^ |
| 57 | 109434738 | F | Chick | 2018 | None recorded | - | - | - |
| 58 | 109434739 | F | Chick | 2018 | None recorded | - | - | - |
| **59** | 109434740 | F | Chick | 2018 | **Same-year siblings: sample 62** | **0.21** | **5** | **S** |
| **60** | 109434741 | M | Chick | 2018 | **Same-year siblings: sample 61** | **0.24** | **6** | **S** |
| **61** | 109434742 | M | Chick | 2018 | **Same-year siblings: sample 60** | **0.24** | **6** | **S** |
| **62** | 109434743 | M | Chick | 2018 | **Same-year siblings: sample 59** | **0.21** | **5** | **S** |
| 64 | 109434745 | F | Chick | 2018 | None recorded | - | - | - |
| **65** | 109434746 | F | Chick | 2018 | **Mother: sample 17** | **0.30** | **2** | **S** |
| 66 | 109434747 | — | Chick | 2018 | None recorded | - | - | - |
| **67** | 109434748 | — | Chick | 2018 | **Same-year siblings: sample 68, sample 69;**  **Different-year siblings: sample 16,** sample 17,  sample 29, sample 32 | **0.12, 0.14;**  **0.13,** -0.01,  0.08, NA | **12, 6;**  **6,** 80,  20, NA | **S, S;**  **S,** R,  I, I |
| **68** | 109434749 | M | Chick | 2018 | **Same-year siblings: sample 67, sample 69;**  **Different-year siblings: sample 16,** sample 17,  sample 29, sample 32 | **0.12, 0.13;**  **0.16,** -0.00,  0.02, NA | **12, 12;**  **12,** 80,  20, NA | **S, S;**  **S,** R,  I, I |
| **69** | 109434750 | — | Chick | 2018 | **Same-year siblings: sample 67, sample 68;**  **Different-year siblings: sample 16,** sample 17,  sample 29, sample 32 | **0.14, 0.13;**  **0.15,** 0.03,  0.09, NA | **6, 12;**  **2,** 80,  20, NA | **S, S;**  **S,** R,  I, I |
| 70 | 106406902 | F | Chick | 2019 | None recorded | - | - | - |
| 71 | 106406903 | F | Chick | 2019 | None recorded | - | - | - |
| **72** | 106406904 | — | Chick | 2019 | **Mother: sample 52;**  Different-year siblings: sample 26 | **0.10;**  0.06 | **2;**  10 | **S;**  I |
| 73 | — | — | Adult | — | None recorded | - | - | - |
| **74** | 106406905 | — | Chick | 2020 | **Father: sample 18;**  **Same-year siblings: sample 75** | **0.17/0.14;**  **0.11/0.18** | **4;**  **4** | **S;**  **S** |
| **75** | 106406907 | M | Chick | 2020 | **Father: sample 18;**  **Same-year siblings: sample 74** | **0.19;**  **0.11/0.18** | **5;**  **4** | **S;**  **S** |
| 76 | 106406908 | — | Chick | 2020 | None recorded | - | - | - |
| 77 | 106406909 | M | Chick | 2020 | Mother: sample 6 | NA | NA | I |
| 78 | 106406910 | — | Chick | 2020 | Same-year siblings: sample 86, sample 87 | 0.06, -0.02 | 80, 80 | I, R^[CBP/EPP]^ |
| **79** | 106406911 | — | Chick | 2020 | **Same-year siblings: sample 84** | **0.20** | **2** | **S** |
| 80 | 106406912 | — | Chick | 2020 | None recorded | - | - | - |
| **81** | 106406913 | — | Chick | 2020 | **Same-year siblings: sample 82, sample 83, sample 85** | **0.14, 0.12, 0.11** | **8, 3, 2** | **S, S, S** |
| **82** | 106406914 | — | Chick | 2020 | **Same-year siblings: sample 81, sample 83, sample 85** | **0.14, 0.18, 0.15** | **8, 8, 8** | **S, S, S** |
| **83** | 106406915 | M | Chick | 2020 | **Same-year siblings: sample 81, sample 82, sample 85** | **0.12, 0.18, 0.16** | **3, 8, 3** | **S, S, S** |
| **84** | 106406916 | F | Chick | 2020 | **Same-year siblings: sample 79** | **0.20** | **2** | **S** |
| **85** | 106406917 | — | Chick | 2020 | **Same-year siblings: sample 81, sample 82, sample 83** | **0.11, 0.15, 0.16** | **8, 8, 3** | **S, S, S** |
| **86** | 106406918 | — | Chick | 2020 | **Same-year siblings:** sample 78, **sample 87** | 0.06, **0.20** | 80, **2** | I, **S** |
| **87** | 106406919 | F | Chick | 2020 | **Same-year siblings:** sample 78, **sample 86** | -0.02, **0.20** | 80, **2** | R^[CBP/EPP]^, **S** |
| 89 | 106406921 | M | Chick | 2021 | None recorded | - | - | - |
| 90 | 106406922 | M | Chick | 2021 | Same-year siblings: sample 91, sample 92 | NA | NA | I, I |
| 91 | 106406923 | — | Chick | 2021 | Same-year siblings: sample 90, sample 92 | NA, 0.01 | NA, 32 | I, I |
| 92 | 106406924 | — | Chick | 2021 | Same-year siblings: sample 90, sample 91 | NA, 0.01 | NA, 32 | I, I |
| **93** | 106406925 | — | Chick | 2021 | **Same-year siblings:** sample 98, **sample 99** | -0.03, **0.30** | 80, **4** | R^[CBP/EPP]^, **S** |
| 94 | 106406926 | M | Chick | 2021 | Same-year siblings: sample 97 | -0.00 | 54 | I |
| **95** | 106406927 | F | Chick | 2021 | **Same-year siblings: sample 96** | **0.36** | **2** | **S** |
| **96** | 106406928 | F | Chick | 2021 | **Same-year siblings: sample 95** | **0.36** | **2** | **S** |
| 97 | 106406929 | — | Chick | 2021 | Same-year siblings: sample 94 | -0.00 | 54 | I |
| 98 | 106406930 | M | Chick | 2021 | Same-year siblings: sample 93, sample 99 | -0.03, -0.05 | 80, 80 | R^[CBP/EPP]^, R^[CBP/EPP]^ |
| **99** | 106406931 | — | Chick | 2021 | **Same-year siblings: sample 93,** sample 98 | **0.30,** -0.05 | **4,** 80 | **S,** R^[CBP/EPP]^ |
| 100 | 106406932 | M | Chick | 2021 | Same-year siblings: sample 102 | NA | NA | I |
| 101 | — | — | Chick | 2021 | None recorded |  |  |  |
| 102 | 106406933 | M | Chick | 2021 | Same-year siblings: sample 100 | NA | NA | I |
| 103 | 106406934 | — | Chick | 2021 | Same-year siblings: sample 104 | -0.04 | 80 | R^[CBP/EPP]^ |
| 104 | 106406935 | — | Chick | 2021 | Same-year siblings: sample 103 | -0.04 | 80 | R^[CBP/EPP]^ |
| 105 | — | — | Chick | 2021 | None recorded |  |  |  |
| 106 | 106406936 | F | Chick | 2021 | None recorded | - | - | - |
| 107 | 106406937 | — | Chick | 2021 | Same-year siblings: sample 110 | -0.01 | 80 | R^[CBP/EPP]^ |
| 108 | 106406938 | — | Chick | 2021 | Same-year siblings: sample 109 | -0.01 | 26 | I |
| 109 | 106406939 | F | Chick | 2021 | Same-year siblings: sample 108 | -0.01 | 26 | I |
| 110 | 106406940 | — | Chick | 2021 | Same-year siblings: sample 107 | -0.01 | 80 | R^[CBP/EPP]^ |
| 111 | 106406941 | — | Chick | 2021 | Father: sample 26; Mother: sample 56 | -0.06; -0.05 | 80, 80 | R^[CBP/EPP]^, R^[CBP]^ |
| 112 | 106406942 | M | Chick | 2021 | None recorded | - | - | - |

**Table S4.** Significant triad (two parents and one offspring) and dyad (one parent and one offspring) outputs following parentage analysis with {apparent} on a breeding population of Hawaiian Stilt (Ae‘o; *Himantopus mexicanus knudsen*) individuals (GD: Gower Dissimilarity metric, GDM: mean GD, GDCV: coefficient of variation among the pairwise GD’s). The central section of the table provides extra information about the individuals based on observed behavioural data. Note that ‘code’ represents the reclassified relationship, depending on hatch year, corrected from the default parent-offspring relationship output by {apparent} (P/O: likely true parent-offspring, FS: likely full siblings, and HS+: half-siblings+ ie. unable to be confirmed as likely true parent-offspring or siblings). The behavioral info column highlights consensus relationships. The right side of the table provides additional genetic information - pairwise genomic relatedness (GR) and relative position in a phylogenetic tree - which together provided a threshold for whether a relationship was supported, rejected, or indeterminate. Values denoted with superscript abbreviations summarize relationships used as evidence for conspecific brood parasitism or extra-pair paternity [CBP/EPP], and inbreeding [IB]. Italicized rows highlight repeat dyads with both parent and offspring roles suggested for each individual.

| **Triads** | | | | | | | | | | | | | |
| --- | --- | --- | --- | --- | --- | --- | --- | --- | --- | --- | --- | --- | --- |
| **Parent 1** | **Parent 2** | **Offspring** | **Cross type** | **SNPs** | **GD** | **p.value** | **Parent birth year** | **Offspring birth year** | **code** | **Behavioral info** | **GR** | **# descendents under most recent common node** | **result** |
| 56 | 26 | 107 | outcross | 4,415 | 0.0408 | 0.0253 | 56: 2018 26: 2015 | 2021 | P/O | Observed mate pair | 56-107: 0.32  26-107: 0.33  56-26:  0.20 | 3 | supported^[IB]^ |
| **Dyads** | | | | | | | | | | | | | |
|  | **Parent** | **Offspring** |  | **GDM p-value** | **GDCV p-value** | **Cumulative**  **p-value** | **Parent birth year** | **Offspring birth year** | **code** | **Behavioral info** | **GR** | **# descendents under most recent common node** | **result** |
|  | 76 | 74 |  | 0.0010 | 0.0009 | 8.32E-07 | 2020 | 2020 | FS |  | 0.10/0.14 | 3 | supported |
|  | 76 | 75 |  | 0.0002 | 0.0059 | 9.46E-07 | 2020 | 2020 | FS |  | 0.19 | 2 | supported |
|  | 62 | 93 |  | 0.0035 | 0.0027 | 9.39E-06 | 2018 | 2021 | P/O |  | 0.29 | 2 | supported |
|  | 57 | 46 |  | 0.0012 | 0.0142 | 1.71E-05 | 2018 | NA | HS+ |  | 0.19 | 7 | supported |
|  | 62 | 59 |  | 0.0008 | 0.2210 | 1.81E-04 | 2018 | 2018 | FS | Observed siblings | 0.21 | 5 | supported |
|  | 62 | 104 |  | 0.0033 | 0.0798 | 2.60E-04 | 2018 | 2021 | P/O |  | 0.24 | 4 | supported |
|  | 82 | 81 |  | 0.0044 | 0.0612 | 2.70E-04 | 2020 | 2020 | FS | Observed siblings | 0.14 | 8 | supported |
|  | 30 | 82 |  | 0.0339 | 0.0100 | 3.38E-04 | 2017 | 2020 | P/O |  | 0.11 | 5 | supported |
|  | 53 | 41 |  | 0.0507 | 0.0139 | 7.07E-04 | NA | 2017 | HS+ |  | 0.07 | 10 | indeterminate |
|  | 70 | 71 |  | 0.0436 | 0.0221 | 9.64E-04 | 2019 | 2019 | FS |  | 0.20 | 2 | supported |
|  | 9 | 66 |  | 0.0087 | 0.1206 | 1.04E-03 | 2016 | 2018 | HS+ |  | 0.16 | 2 | supported |
|  | *66* | *9* |  | *0.0296* | *0.0478* | *1.41E-03* | *2018* | *2016* | *Repeat* | | *Repeat* | | |
|  | 58 | 29 |  | 0.0494 | 0.0288 | 1.42E-03 | 2018 | 2017 | HS+ |  | 0.17 | 2 | supported |
|  | 17 | 65 |  | 0.0209 | 0.0921 | 1.92E-03 | 2016 | 2018 | P/O | Observed parent-offspring | 0.30 | 2 | supported |
|  | 53 | 49 |  | 0.0700 | 0.0446 | 3.12E-03 | NA | 2017 | HS+ |  | 0.08 | 2 | indeterminate |
|  | *41* | *53* |  | *0.0378* | *0.0936* | *3.54E-03* | *2017* | *NA* | *Repeat* | | *Repeat* | | |
|  | 69 | 16 |  | 0.0467 | 0.1071 | 5.00E-03 | 2018 | 2016 | HS+ | Observed siblings | 0.15 | 2 | supported |
|  | 87 | 58 |  | 0.0343 | 0.1629 | 5.59E-03 | 2020 | 2018 | HS+ |  | 0.23 | 4 | supported |
|  | 34 | 111 |  | 0.0455 | 0.1261 | 5.75E-03 | 2017 | 2021 | P/O |  | 0 | 80 | rejected^[CBP/EPP]^ |
|  | 58 | 87 |  | *0.0598* | *0.0979* | *5.86E-03* | 2018 | 2020 | *Repeat* | | *Repeat* | | |
|  | 69 | 67 |  | 0.0488 | 0.1388 | 6.78E-03 | 2018 | 2018 | FS | Observed siblings | 0.14 | 6 | supported |
|  | 87 | 86 |  | 0.0506 | 0.1458 | 7.37E-03 | 2020 | 2020 | FS | Observed siblings | 0.20 | 2 | supported |
|  | 53 | 106 |  | 0.1992 | 0.0516 | 1.03E-02 | NA | 2021 | HS+ |  | 0.01 | 80 | rejected |

Supplementary Figures

**Figure S1.** Distribution of pairwise genomic relatedness values for a filtered dataset of 81 Hawaiian Stilt (Ae‘o; *Himantopus mexicanus knudsen*) samples, calculated using Yang’s A_jk_ estimator in VCFtools.

Figure S2. Maximum clade credibility phylogenetic tree from BEAST analysis of Hawaiian Stilt (Ae‘o; *Himantopus mexicanus knudsen*) samples, summarized by treeannotator and visualized with the {ggtree} R package. Tip labels represent individual bird sample IDs and indicate sex where data are available.
